# Essential updates 2020/2021: Current topics of simulation and navigation in hepatectomy

**DOI:** 10.1002/ags3.12542

**Published:** 2021-12-23

**Authors:** Yu Saito, Mitsuo Shimada, Yuji Morine, Shinichiro Yamada, Maki Sugimoto

**Affiliations:** ^1^ Department of Surgery Tokushima University Tokushima Japan; ^2^ Okinaga Research Institute Teikyo University Chiyoda‐ku Japan

**Keywords:** artificial intelligence, extended reality, ICG fluorescence, liver surgery, navigation, simulation

## Abstract

With the development of three‐dimensional (3D) simulation software, preoperative simulation technology is almost completely established. The remaining issue is how to recognize anatomy three‐dimensionally. Extended reality is a newly developed technology with several merits for surgical application: no requirement for a sterilized display monitor, better spatial awareness, and the ability to share 3D images among all surgeons. Various technology or devices for intraoperative navigation have also been developed to support the safety and certainty of liver surgery. Consensus recommendations regarding indocyanine green fluorescence were determined in 2021. Extended reality has also been applied to intraoperative navigation, and artificial intelligence (AI) is one of the topics of real‐time navigation. AI might overcome the problem of liver deformity with automatic registration. Including the issues described above, this article focuses on recent advances in simulation and navigation in liver surgery from 2020 to 2021.

## 1 INTRODUCTION

Advances in perioperative care and surgical techniques have significantly improved the outcomes of liver resection during the last three decades. Liver surgery has inherent challenges, including difficult anticipation of complex and variable intrahepatic anatomy and the need for cognitive analysis by the surgeon to integrate preoperative imaging information into the operative field. Therefore, simulation and navigation techniques have been developed in this field.

In preoperative simulation, three‐dimensional (3D) simulation technology was developed in Germany in the early 2000s,^1^ and immediately thereafter software based on an original algorithm was developed in Japan. The development of intraoperative navigation techniques may also help surgeons to perform liver resections as planned. Intraoperative navigation began with intraoperative ultrasound (US) in 1980^2^ and progressed to virtual hepatectomy (Hx),^3^, ^4^, ^5^, ^6^, ^7^ real‐time virtual sonography,^8^, ^9^, ^10^, ^11^, ^12^ and finally indocyanine green (ICG) fluorescence.^13^, ^14^, ^15^, ^16^, ^17^, ^18^, ^19^, ^20^ Thus, intraoperative navigation has gradually evolved during the past 40 y.

This biannual review discusses the essential updates to simulation and navigation in Hx that occurred in the 2‐y period from 2020 to 2021.

## 2 PREOPERATIVE SIMULATION

Preoperative 3D simulation has enabled surgeons to obtain a great deal of information, such as detailed anatomical visualization, the precise volume of each segment and each hepatic venous drainage area, and prediction of postoperative liver failure (POLF). As a result, more aggressive and complicated surgeries can be safely performed. We herein summarize the recent refinements of preoperative simulation in liver surgery from 2020 to 2021 (Table 1).

**TABLE 1:** Preoperative simulation in liver surgery

Author	Year	Category	Article type/Patients’ number	Information
Ozer^21^	2021	Anatomical visualization	A case study (n = 5) Questionnaire (n = 22)	3D printing porta‑celiac vascular model Surgical plan for resident trainees in Hx
Kuroda^22^	2020	Anatomical visualization	A case study (n = 5) Comparison of surgical outcomes (n = 212)	3D printing liver model Vessel's simulation in donor Hx of LDLT
Larghi^24^ Laureiro^23^	2020	Anatomical visualization	A case study (n = 1)	3D printing liver model Surgical plan in hilar cholangiocarcinoma
Huettl^25^	2021	Anatomical visualization	A case study (n = 20) Questionnaire (n = 20)	VR liver Preoperative visualization of vessels in Hx
Boedecker^26^	2021	Anatomical visualization	A case study (n = 1)	VR (immersive into liver) Surgical plan/Clinical presentation in Hx
Pelanis^27^	2020	Anatomical visualization	A case study (n = 1) Questionnaire (n = 28)	MR liver Preoperative visualization of vessels in Hx
Saito^28^	2020	Volumetry	Retrospective single‐center study (n = 66)	Hx based on hybrid concept of portal perfusion and venous drainage area
Li^29^	2021	Volumetry	Retrospective single‐center study (n = 102)	Simulation of portal or venous associated remnant liver ischemia or congestion
Procopio^30^	2021	Prediction of POLF	Retrospective single‐center study (n = 30)	Volumetry using 3D simulation
Araki^32^	2020	Prediction of POLF	Retrospective single‐center study (n = 155)	SI in remnant liver with ROB‐MRI
Notake^33^	2021	Prediction of POLF	Retrospective single‐center study (n = 67)	SI in remnant liver with ROB‐MRI

Abbreviations: EOB‐MRI, ethoxybenzyl‐magnetic; Hx, hepatectomy; LDLT, living‐donor liver transplantation; MR, mixed reality; SI, signal intensity; VR, virtual reality.

### 2.1 Anatomical visualization: 3D printing liver and extended reality

If a 3D liver model including the tumor, each vessel, and the liver parenchyma is created, it is meaningless to display that model on a 2D monitor or printed paper because of the lack of spatial awareness. Therefore, many reports have described the usefulness of 3D printing of liver models for operative planning or medical education.^21^, ^22^, ^23^ Because 3D printing of the liver results in a model of the patient's own liver, accurate information can be obtained regarding the vessel anatomy, the relationship between the tumor and vessels, and the parenchymal cutting plane. Another advantage of 3D printing is that the operator can freely pick up the patient's own liver. The material used for 3D printing is also being developed in various ways. However, the high cost and complexity of the creation process are undeniable.

Recently, new technologies involving virtual reality (VR), augmented reality (AR), and mixed reality (MR), all of which can be referred to as extended reality (XR), have been developed and applied to various operative simulations. Head mount displays (HMDs) intrinsically provide the user with an egocentric viewpoint and allow the user to work hands‐free without a monitor. Especially in VR, the surgeons can be immersed in the patient's own liver. The merits of the application of XR techniques to surgical support include no need for a sterilized display monitor, better spatial awareness, and the ability to share 3D images among all surgeons.^24^ XR techniques are applied to preoperative planning or visualization of vessels in liver surgery,^25^, ^26^, ^27^ and XR images are suitable for clinical presentation because of their sharing function. Huettl et al^25^ compared 3D printed liver models and VR liver models and concluded that 3D VR liver models enable a better and partially faster anatomical orientation than 3D printed liver models. XR technology is still in its early stages. HMDs should be refined into lighter, simpler, and easier to operate devices.

### 2.2 Volumetry: Portal perfusion and venous drainage

Preoperative volumetry is essential to ensuring safe hepatic resection by estimating the volume of both the portal perfusion and venous drainage area. In 2020–2021, Saito et al^28^ proposed Hx based on a hybrid concept of the portal perfusion of the anterior segment and venous drainage area of the superior right hepatic vein. The perfusion area of the anterior segment crossed over the superior right hepatic vein in one‐fourth of the patients in the study. The authors considered that less invasive Hx based on a hybrid concept might be an alternative to right Hx. Li et al^29^ preoperatively simulated portal or hepatic vein‐associated remnant liver ischemia or congestion, and it led to postoperative complications.

### 2.3 Prediction of POLF

Aside from 3D reconstruction or simulation software, also simple liver function simulation is also critical in liver surgery. Preoperative volumetry can estimate the remnant liver volume and predict POLF.^30^ Gadolinium ethoxybenzyl diethylenetriamine pentaacetic acid (Gd‐EOB‐DTPA)‐enhanced magnetic resonance imaging (EOB‐MRI) can also be used to evaluate liver functional reserve.^31^ Functional remnant liver volumetry with signal intensity in EOB‐MRI can precisely predict POLF of Hx involving more than one segment.^32^ EOB‐MRI is also useful for predicting POLF after major Hx for biliary malignancy.^33^ In terms of a “one‐stop shop” of preoperative simulation, EOB‐MRI may be the most useful modality for detecting tumors, simulating vessel anatomy, and estimating remnant functional reserve.

## 3 INTRAOPERATIVE NAVIGATION

Navigation in liver surgery began in 1985 when Makuuchi et al^2^ performed anatomical resection with dye staining using intraoperative US. Various medical devices have been developed to support the safety and certainty of liver surgery with recent advances in medical engineering technology.

Fluorescent navigation using ICG has been clinically applied in various ways for liver navigation surgery. As described above for preoperative simulation, XR techniques have also been applied to intraoperative support systems. Furthermore, artificial intelligence (AI) technology has been introduced to intraoperative navigation. We herein summarize the recent refinements of intraoperative navigation in liver surgery from 2020 to 2021.

### 3.1 ICG staining

Indocyanine green emits a fluorescent wavelength and is clearly visualized when irradiated with near‐infrared light (760 nm). In total, 73 articles were found in PubMed in 2020–2021 using the search terms “ICG” and “Liver surgery”; articles describing the intraoperative use of ICG were more limited and can be classified into liver area staining or tumor detection. Recently, ICG has also been widely used in laparoscopic surgery (Table 2).

**TABLE 2:** ICG navigation in liver surgery

Author	Year	Category	Article type/Patients’ number	Information
Wang^34^	2021	Area staining/Tumor detection	Guideline	Recommendation Class; IIa or IIb Evidence level; II‐2 or II‐3
Lu^35^	2021	Area staining	Retrospective single‐center study (n = 120)	Better short‐term outcomes and surgical margin
Kim^36^	2021	Area staining	Retrospective single‐center study (n = 76)	Laparoscopic donor's Hx Demarcating exact midplane
Marino^37^	2020	Area staining	Retrospective single‐center study (n = 40) Positive (n = 20) and Negative (20) staining	Robotic‐assisted Hx
He^38^	2020	Area staining	A randomized controlled trial (n = 46)	Hx for hepatolithiasis Better short‐term outcomes
Kubo^39^	2020	Area staining	Retrospective single‐center study (n = 12)	Determining areas of liver congestion of RHV
Zhang^40^	2020	Area staining	Retrospective single‐center study (n = 64)	Collaboration with preoperative 3D simulation and intraoperative ICG
Xu^41^	2020	Area staining	Retrospective single‐center study (n = 36)	Technical difficulty in positive staining (Success rate; around 50%)
Aoki^42^	2020	Area staining	A case study (n = 14)	Preoperative positive percutaneous staining before laparoscopic surgery
Lim^43^	2021	Tumor detection	Retrospective single‐center study (n = 32)	Detection of intrahepatic tumors
Yamamura^44^	2020	Tumor detection	A case study (n = 1)	Detection of extrahepatic tumors
Hayashi^45^	2021	Tumor detection	A case study (n = 1)	Detection of extrahepatic tumors
Tashiro^46^	2020	Tumor detection	Retrospective single‐center study (n = 125)	Better surgical margin
Purich^47^	2020	Prediction of POLF	A systematic review and meta‐analysis	overall sensitivity; 0.75 Additional tumor detection; 11.6%

Abbreviation: RHV, right hepatic vein.

In 2021, consensus recommendations were established for the use of fluorescence imaging with ICG in hepatobiliary surgery.^34^ Seven recommendations were formulated. In area staining, the consensus states that “ICG is helpful in delineating segmental boundaries in both open and minimally invasive liver resection (Recommendation Class IIa/IIb).” In tumor imaging, the consensus states that “ICG is helpful to localize subcapsular tumors within 8 mm of the liver surface or cut surface of the liver parenchyma, and may reduce the risk of positive margins (Recommendation Class IIa/IIb).”

The usefulness of positive and negative staining of each segment has been fully reported. In a retrospective single‐center study of 120 cases, Lu et al^35^ reported that ICG staining contributed to a shorter operative time and lower amount of intraoperative blood loss and that it helped to achieve a wide surgical margin. Furthermore, ICG staining was performed in special types of Hx, such as laparoscopic donor Hx,^36^ robotic Hx,^37^ and Hx for hepatolithiasis.^38^ Kubo et al^39^ used ICG staining for navigation of the venous drainage area of the right hepatic vein. Collaboration with a preoperative 3D simulation modality and intraoperative ICG staining with AR techniques was also introduced in 30 patients undergoing laparoscopic Hx.^40^ This new navigation technology contributed to better surgical outcomes; however, its effect on the long‐term prognosis remains unclear. In terms of the technical performance of ICG staining, although negative staining is relatively easy, positive staining is sometimes difficult, depending on the location of the tumors, especially in laparoscopic Hx. Xu et al^41^ described their failed cases of positive staining, reporting a success rate of around 50%. Performing positive staining requires the surgeon to be proficient in intraoperative laparoscopic US (LUS), comfortable performing US‐guided puncture, and skillful in interpreting the preoperative 3D image simulation. The manipulation of LUS is the most demanding part. Therefore, a new kind of LUS probe should be developed for useful positive staining in the future. Because LUS is difficult, Aoki et al^42^ performed US‐guided preoperative positive percutaneous staining immediately before laparoscopic surgery. This was a very simple technique and may be a reasonable way to resolve the technical difficulty of the procedure.

In tumor detection, ICG is useful to identify not only intrahepatic tumors^43^ but also extrahepatic metastatic tumors such as those in the adrenal gland^44^ or abdominal wall.^45^ Tumor detection with ICG contributes to the safe achievement of surgical margins during liver resection.^46^ Purich et al^47^ performed a systematic review and meta‐analysis of the diagnostic test accuracy of ICG. The sensitivity of intraoperative ICG‐related imaging for superficial tumors was high; however, the overall sensitivity was low, at 0.75, suggesting that this technique would have to be used in combination with current identification methods such as intraoperative US. Their study also showed that intraoperative ICG fluorescence imaging was able to detect additional malignant hepatic tumors in 11.6% of patients.

### 3.2 XR

In XR techniques, VR is useful for preoperative simulation, allowing the surgeon to become immersed in the patient's own liver with better spatial awareness. AR or MR techniques should be used with intraoperative navigation tools because surgeons must examine the real operative field in both open and laparoscopic surgery.

In total, 27 articles were found in PubMed in 2020–2021 using the search terms “VR/AR/MR” and “Liver surgery” (Table 3). Most of these reports focused on AR‐guided navigation. A preoperatively reconstructed 3D liver model should be overlaid onto the real liver. Unlike for neurosurgery, otolaryngology, and orthopedic surgery, in which rigid structures facilitate a rather unproblematic registration, liver surgery is associated with the problem of deformation of abdominal tissues and organs. This deformation results in a difficult registration procedure, potentially requiring nonrigid registration techniques to achieve sufficient registration accuracy. Therefore, various ways to perform registration of a preoperative 3D liver model have been developed.

**TABLE 3:** XR navigation in liver surgery

Author	Year	Category	Article type/Patients’ number	Information
Golse^48^	2021	AR overlay	A case study (n = 5)	Real‐time marker less registration with RGB‐D camera
Espinel^49^	2020	AR overlay	A case study (n = 7)	Laparoscope and liver surface as landmark Average registration error <1.0 cm
Prevost^50^	2020	AR overlay	A case study (n = 10)	Laparoscope and 4 points as landmark Mean fiducial registration error 14 mm
Bertrand^51^	2020	AR overlay	A case study (n = 17)	“Hepataug system” Safety and feasibility
Pelanis^52^	2021	AR overlay	A case study (n = 4)	Cone beam CT / Optical tracking system Mean target registration error 3.8 mm
Zhang^53^	2020	AR overlay	Retrospective single‐center study (n = 85)	AR contributed to less blood loss and shorter hospital stays
Saito^23^	2020	MR hologram	A case study (n = 2)	Last‐minute simulation before Gliisonean pedicle approach
Saito^55^	2021	MR hologram	A case study (n = 2)	Anatomy understanding of intrahepatic anatomy especially in B1 origination
Aoki^56^	2020	MR hologram	A case study (n = 1)	Holography‐guided percutaneous puncture in positive staining

Abbreviations: AR, augmented reality; CT, computed tomography; RGB, right green blue.

Golse et al^48^ placed a special camera called the e RGB‐D camera in the operation room to perform real‐time markerless registration in open liver surgery. In laparoscopic surgery, combination techniques with intraoperative calibration of the laparoscope and various landmarks to define the liver anatomy such as the falciform ligament, edge of the liver, and gall bladder are commonly used.^49^, ^50^, ^51^ Pelanis et al^52^ performed intraoperative cone‐beam computed tomography (CT) and used an optical tracking system in registration. Target registration error and fiducial registration error were evaluated in those reports^49^, ^50^, ^51^, ^52^ and ranged from 3.0–14.0 mm. Such an AR overlay navigation system contributed to a reduction in vascular injury and more rapid postoperative recovery.^53^ Therefore, more refinements of accurate registration should be implemented in the future.

Mixed reality techniques with 3D computer‐generated models called holograms have also been introduced intraoperatively with HMDs. Saito et al used a hologram based on preoperative CT immediately before performing the Glissonean pedicle approach in Hx^54^ and a hologram based on intraoperative cholangiography immediately before dissecting the intrahepatic bile duct in biliary surgery.^55^ Operators and assistants can share the same hologram from each angle with HMDs and observe the detailed biliary anatomy around the dissected bile duct (Video S1). A system called a “virtual session” was also recently introduced. Conductor (operator), two assistants and a remote participant, who is not in the operating room, can share the hologram in the metaverse. Conductor explains the operative plan to assistants and the remote participant. We plan to apply this system in the field of remote medical care in the near future (Video S2). Strictly speaking, holograms contribute to “last‐minute simulation,” not navigation. However, the hologram might be a new next‐generation operation‐support tool in terms of spatial awareness, sharing, and simplicity. Aoki et al^56^ also reported holography‐guided percutaneous puncture in positive staining with ICG in laparoscopic surgery. As described in the ICG navigation section, positive staining especially in laparoscopic Hx is sometimes difficult, depending on the location of the tumors. This holographic guidance might help the operator to develop a better imagination.

### 3.3 AI

Artificial intelligence technology was recently introduced to surgical navigation. AI should provide image recognition, focusing on anatomical structures, image recognition focusing on the surgical procedure itself, and control against incorrect performance of the surgical procedure. AI can already reportedly recognize the surgical process,^57^ surgical instruments such as laparoscopic forceps,^58^ and anatomical landmarks^59^, ^60^ in cholecystectomy or colorectal surgery. AI has also been applied to assessment of surgical skill.^61^

Nazir et al^62^ reported a new searching and tagging system that recognizes various anatomical landmarks in laparoscopic liver surgery. Only one article focused on intraoperative navigation with AI in liver surgery from 2020–2021. AI is not yet frequently used in liver surgery, but future technological applications are expected.

To date, AI has only been used for the recognition of anatomical structures based on information of surgical field images. In the future, some suggestions or attention on anatomical structure information that cannot be directly seen in the surgical field are expected. Furthermore, an integrated analysis of real surgical field images and preoperative modalities should be developed for AI navigation surgery.

## 4 CONCLUSION

The current status of simulation and navigation in hepatectomy from 2020 to 2021 has been reviewed. Preoperative simulation technology is already almost fully established; the next step is navigation in liver surgery. ICG staining is now widely used for area staining and tumor detection. Some refinements should be developed in terms of positive staining in laparoscopic liver surgery. XR techniques provide amazing new information regarding the liver anatomy, with better spatial awareness; however, the problems of registration and real‐time liver deformity remain to be solved. Finally, the development of AI technology is ongoing. The establishment of various simulation and navigation technologies should help surgeons to perform safer liver resection.

## DISCLOSURE

Conflict of interest: All authors declare that they have no competing interests.

## Supporting information

Video S1

Video S2

## References

[1] Selle D , Preim B , Schenk A , Peitgen HO . Analysis of vasculature for liver surgical planning. IEEE Trans Med Imaging. 2002;21:1344–57. 12575871 10.1109/TMI.2002.801166

[2] Makuuchi M , Hasegawa H , Yamazaki S . Ultrasonically guided subsegmentectomy. Surg Gynecol Obstet. 1985;161:346–50. 2996162

[3] Oshiro Y , Yano H , Mitani J , Kim S , Kim J , Fukunaga K , et al. Novel 3‐dimensional virtual hepatectomy simulation combined with real‐time deformation. World J Gastroenterol. 2015;21(34):9982–92. 26379403 10.3748/wjg.v21.i34.9982 PMC4566391

[4] Oshiro Y , Ohkohchi N . Three‐dimensional liver surgery simulation: computer‐assisted surgical planning with three‐dimensional simulation software and three‐dimensional printing. Tissue Eng Part A. 2017;23(11–12):474–80. 28343411 10.1089/ten.TEA.2016.0528

[5] Mise Y , Tani K , Aoki T , Sakamoto Y , Hasegawa K , Sugawara Y , et al. Virtual liver resection: computer‐assisted operation planning using a three‐dimensional liver representation. Kokudo N J Hepatobiliary Pancreat Sci. 2013;20(2):157–64. 23135735 10.1007/s00534-012-0574-y

[6] Mise Y , Hasegawa K , Satou S , Shindoh J , Miki K , Akamatsu N , et al. How has virtual hepatectomy changed the practice of liver surgery?: Experience of 1194 virtual hepatectomy before liver resection and living donor liver transplantation. Ann Surg. 2018;268(1):127–33. 28288065 10.1097/SLA.0000000000002213

[7] Aoki T , Murakami M , Koizumi T , Fujimori A , Gareer H , Enami Y , et al. Three‐dimensional virtual endoscopy for laparoscopic and thoracoscopic liver resection. J Am Coll Surg. 2015;221(2):e21–6. 26206658 10.1016/j.jamcollsurg.2015.04.012

[8] Sandulescu L , Saftoiu A , Dumitrescu D , Ciurea T . The role of real‐time contrast‐enhanced and real‐time virtual sonography in the assessment of malignant liver lesions. J Gastrointestin Liver Dis. 2009;18(1):103–8. 19337645

[9] Kasuya K , Sugimoto K , Kyo B , Nagakawa Y , Ikeda T , Mori Y , et al. Ultrasonography‐guided hepatic tumor resection using a real‐time virtual sonography with indocyanine green navigation (with videos). J Hepatobiliary Pancreat Sci. 2011;18(3):380–5. 21127911 10.1007/s00534-010-0356-3

[10] Satou S , Aoki T , Kaneko J , Sakamoto Y , Hasegawa K , Sugawara Y , et al. Initial experience of intraoperative three‐dimensional navigation for liver resection using real‐time virtual sonography. Surgery. 2014;155(2):255–62. 24579091 10.1016/j.surg.2013.08.009

[11] Takamoto T , Mise Y , Satou S , Kobayashi Y , Miura K , Saiura A , et al. Feasibility of intraoperative navigation for liver resection using real‐time virtual sonography with novel automatic registration system. World J Surg. 2018;42(3):841–8. 28879512 10.1007/s00268-017-4210-5

[12] Lv A , Li Y , Qian HG , Qiu H , Hao CY . Precise navigation of the surgical plane with intraoperative real‐time virtual sonography and 3D simulation in liver resection. J Gastrointest Surg. 2018;22(10):1814–8. 30039451 10.1007/s11605-018-3872-0

[13] Aoki T , Yasuda D , Shimizu Y , Odaira M , Niiya T , Kusano T , et al. Image‐guided liver mapping using fluorescence navigation system with indocyanine green for anatomical hepatic resection. World J Surg. 2008;32(8):1763–7. 18543027 10.1007/s00268-008-9620-y

[14] Aoki T , Murakami M , Koizumi T , Kusano T , Fujimori A , Enami Y , et al. Preoperative tattooing for precise and expedient localization of landmark in laparoscopic liver resection. J Am Coll Surg. 2015;221(5):e97–e101. 26278038 10.1016/j.jamcollsurg.2015.07.444

[15] Ishizawa T , Fukushima N , Shibahara J , Masuda K , Tamura S , Aoki T , et al. Real‐time identification of liver cancers by using indocyanine green fluorescent imaging. Cancer. 2009;115:2491–504. 19326450 10.1002/cncr.24291

[16] Miyata A , Ishizawa T , Tani K , Shimizu A , Kaneko J , Aoki T , et al. Reappraisal of a dye‐staining technique for anatomic hepatectomy by the concomitant use of indocyanine green fluorescence imaging. J Am Coll Surg. 2015;221:e27–36. 26206659 10.1016/j.jamcollsurg.2015.05.005

[17] Gotoh K , Yamada T , Ishikawa O , Takahashi H , Eguchi H , Yano M , et al. A novel image‐guided surgery of hepatocellular carcinoma by indocyanine green fluorescence imaging navigation. J Surg Oncol. 2009;100:75–9. 19301311 10.1002/jso.21272

[18] Kobayashi Y , Kawaguchi Y , Kobayashi K , Mori K , Arita J , Sakamoto Y , et al. Portal vein territory identification using indocyanine green fluorescence imaging: technical details and short‐term outcomes. J Surg Oncol. 2017;116:921–31. 28695566 10.1002/jso.24752

[19] van der Vorst JR , Schaafsma BE , Hutteman M , Verbeek FP , Liefers GJ , Hartgrink HH , et al. Near‐infrared fluorescence‐guided resection of colorectal liver metastases. Cancer. 2013;119:3411–8. 23794086 10.1002/cncr.28203 PMC3775857

[20] Nishino H , Hatano E , Seo S , Nitta T , Saito T , Nakamura M , et al. Real‐time navigation for liver surgery using projection mapping with indocyanine green fluorescence: development of the novel medical imaging projection system. Ann Surg. 2018;267(6):1134–40. 28181939 10.1097/SLA.0000000000002172

[21] Ozer MA , Uguz A , Unalp OV , Coker A , Govsa F , Guler E , et al. Perceptions of porta‐celiac vascular models for hepatic surgery and their use in residency training. Surg Radiol Anat. 2021;43(8):1359–71. 33677685 10.1007/s00276-021-02724-7

[22] Kuroda S , Kihara T , Akita Y , Kobayashi T , Nikawa H , Ohdan H . Simulation and navigation of living donor hepatectomy using a unique three‐dimensional printed liver model with soft and transparent parenchyma. Surg Today. 2020;50(3):307–13. 31471747 10.1007/s00595-019-01868-9

[23] Saito Y , Sugimoto M , Imura S , Morine Y , Ikemoto T , Iwahashi S , et al. Intraoperative 3D hologram support with mixed reality techniques in liver surgery. Ann Surg. 2020;271(1):e4–7. 31425293 10.1097/SLA.0000000000003552

[24] Larghi Laureiro Z , Novelli S , Lai Q , Mennini G , D'andrea V , Gaudenzi P , et al. There is a great future in plastics: personalized approach to the management of hilar cholangiocarcinoma using a 3‐D‐printed liver model. Dig Dis Sci. 2020;65(8):2210–5. 32440740 10.1007/s10620-020-06326-y

[25] Huettl F , Saalfeld P , Hansen C , Preim B , Poplawski A , Kneist W , et al. Virtual reality and 3D printing improve preoperative visualization of 3D liver reconstructions‐results from a preclinical comparison of presentation modalities and user's preference. Ann Transl Med. 2021;9(13):1074. 34422986 10.21037/atm-21-512 PMC8339861

[26] Boedecker C , Huettl F , Saalfeld P , Paschold M , Kneist W , Baumgart J , et al. Using virtual 3D‐models in surgical planning: workflow of an immersive virtual reality application in liver surgery. Langenbecks Arch Surg. 2021;406(3):911–5. 33710462 10.1007/s00423-021-02127-7 PMC8106601

[27] Pelanis E , Kumar RP , Aghayan DL , Palomar R , Fretland ÅA , Brun H , et al. Use of mixed reality for improved spatial understanding of liver anatomy. Minim Invasive Ther Allied Technol. 2020;29(3):154–60. 31116053 10.1080/13645706.2019.1616558

[28] Saito Y , Imura S , Morine Y , Ikemoto T , Yamada S , Shimada M . A hepatectomy based on a hybrid concept of portal perfusion of anterior segment and venous drainage area of superior right hepatic vein. Am Surg. 2020;3134820984872. Online ahead of print. 33382339 10.1177/0003134820984872

[29] Li XL , Xu B , Zhu XD , Huang C , Shi GM , Shen YH , et al. Simulation of portal/hepatic vein associated remnant liver ischemia/congestion by three‐dimensional visualization technology based on preoperative CT scan. Ann Transl Med. 2021;9(9):756. 34268369 10.21037/atm-20-7920 PMC8246180

[30] Procopio F , Cimino M , Viganò L , Colombo AE , Franchi E , Costa G , et al. Prediction of remnant liver volume using 3D simulation software in patients undergoing R1vasc parenchyma‐sparing hepatectomy for multiple bilobar colorectal liver metastases: reliability, clinical impact, and learning curve. HPB (Oxford). 2021;23(7):1084–94. 33353822 10.1016/j.hpb.2020.11.005

[31] Yamada S , Shimada M , Morine Y , Imura S , Ikemoto T , Saito Y , et al. A new formula to calculate the resection limit in hepatectomy based on Gd‐EOB‐DTPA‐enhanced magnetic resonance imaging. PLoS One. 2019;14(1):e0210579. 30682046 10.1371/journal.pone.0210579 PMC6347147

[32] Araki K , Harimoto N , Kubo N , Watanabe A , Igarashi T , Tsukagoshi M , et al. Functional remnant liver volumetry using Gd‐EOB‐DTPA‐enhanced magnetic resonance imaging (MRI) predicts post‐hepatectomy liver failure in resection of more than one segment. HPB (Oxford). 2020;22(2):318–27. 31477460 10.1016/j.hpb.2019.08.002

[33] Notake T , Shimizu A , Kubota K , Ikehara T , Hayashi H , Yasukawa K , et al. Hepatocellular uptake index obtained with gadoxetate disodium‐enhanced magnetic resonance imaging in the assessment future liver remnant function after major hepatectomy for biliary malignancy. BJS Open. 2021;5(4):zraa048. 34254117 10.1093/bjsopen/zraa048 PMC8275880

[34] Wang X , Teh CSC , Ishizawa T , Aoki T , Cavallucci D , Lee SY , et al. Consensus guidelines for the use of fluorescence imaging in hepatobiliary surgery. Ann Surg. 2021;274(1):97–106. 33351457 10.1097/SLA.0000000000004718

[35] Lu H , Gu J , Qian XF , Dai XZ . Indocyanine green fluorescence navigation in laparoscopic hepatectomy: a retrospective single‐center study of 120 cases. Surg Today. 2021;51(5):695–702. 33128594 10.1007/s00595-020-02163-8 PMC8055570

[36] Kim J , Hong SK , Lim J , Lee JM , Cho JH , Choi Y , et al. Demarcating the exact midplane of the liver using indocyanine green near‐infrared fluorescence imaging during laparoscopic donor hepatectomy. Liver Transpl. 2021;27(6):830–9. 33583130 10.1002/lt.26019

[37] Marino MV , Podda M , Fernandez CC , Ruiz MG , Fleitas MG . The application of indocyanine green‐fluorescence imaging during robotic‐assisted liver resection for malignant tumors: a single‐arm feasibility cohort study. HPB (Oxford). 2020;22(3):422–31. 31409539 10.1016/j.hpb.2019.07.013

[38] He K , Hong X , Chi C , Cai C , Wang K , Li P , et al. A new method of near‐infrared fluorescence image‐guided hepatectomy for patients with hepatolithiasis: a randomized controlled trial. Surg Endosc. 2020;34(11):4975–82. 32020287 10.1007/s00464-019-07290-z

[39] Kubo N , Araki K , Harimoto N , Ishii N , Tsukagoshi M , Igarashi T , et al. Hepatic resection for the right hepatic vein drainage area with indocyanine green fluorescent imaging navigation. J Hepatobiliary Pancreat Sci. 2020;27(7):371–9. 32068353 10.1002/jhbp.728

[40] Zhang P , Luo H , Zhu W , Yang J , Zeng N , Fan Y , et al. Real‐time navigation for laparoscopic hepatectomy using image fusion of preoperative 3D surgical plan and intraoperative indocyanine green fluorescence imaging. Surg Endosc. 2020;34(8):3449–59. 31705286 10.1007/s00464-019-07121-1

[41] Xu Y , Chen M , Meng X , Lu P , Wang X , Zhang W , et al. Laparoscopic anatomical liver resection guided by real‐time indocyanine green fluorescence imaging: experience and lessons learned from the initial series in a single center. Surg Endosc. 2020;34(10):4683–91. 32500459 10.1007/s00464-020-07691-5

[42] Aoki T , Koizumi T , Mansour DA , Fujimori A , Kusano T , Matsuda K , et al. Ultrasound‐guided preoperative positive percutaneous indocyanine green fluorescence staining for laparoscopic anatomical liver resection. J Am Coll Surg. 2020;230(3):e7–e12. 31756381 10.1016/j.jamcollsurg.2019.11.004

[43] Lim HJ , Chiow AKH , Lee LS , Tan SS , Goh BK , Koh YX , et al. Novel method of intraoperative liver tumour localisation with indocyanine green and near‐i nfrared imaging. Singapore Med J. 2021;62(4):182–9. 31680180 10.11622/smedj.2019137 PMC8801829

[44] Yamamura K , Beppu T , Sato N , Kinoshita K , Oda E , Yuki H , et al. Complete removal of adrenal metastasis in hepatocellular carcinoma using indocyanine green fluorescent imaging. Anticancer Res. 2020;40(10):5823–8. 32988911 10.21873/anticanres.14600

[45] Hayashi H , Shimizu A , Motoyama H , Kubota K , Notake T , Sugenoya S , et al. Usefulness and limitation of indocyanine green fluorescence for detection of peritoneal recurrence after hepatectomy for hepatocellular carcinoma: a case report. BMC Surg. 2021;21(1):107. 33653302 10.1186/s12893-021-01111-8 PMC7923305

[46] Tashiro Y , Aoki T , Hirai T , Koizumi T , Mansou DA , Kusano T , et al. Pathological validity of using near‐infrared fluorescence imaging for securing surgical margins during liver resection. Anticancer Res. 2020;40(7):3873–82. 32620627 10.21873/anticanres.14377

[47] Purich K , Dang JT , Poonja A , Sun WYL , Bigam D , Birch D , et al. Intraoperative fluorescence imaging with indocyanine green in hepatic resection for malignancy: a systematic review and meta‐analysis of diagnostic test accuracy studies. Surg Endosc. 2020;34(7):2891–903. 32266547 10.1007/s00464-020-07543-2

[48] Golse N , Petit A , Lewin M , Vibert E , Cotin S . Augmented reality during open liver surgery using a markerless non‐rigid registration system. J Gastrointest Surg. 2021;25(3):662–71. 32040812 10.1007/s11605-020-04519-4

[49] Espinel Y , Özgür E , Calvet L , Le Roy B , Buc E , Bartoli A . Combining visual cues with interactions for 3D–2D registration in liver laparoscopy. Ann Biomed Eng. 2020;48(6):1712–27. 32112344 10.1007/s10439-020-02479-z

[50] Prevost GA , Eigl B , Paolucci I , Rudolph T , Peterhans M , Weber S , et al. Efficiency, accuracy and clinical applicability of a new image‐guided surgery system in 3D laparoscopic liver surgery. J Gastrointest Surg. 2020;24(10):2251–8. 31621024 10.1007/s11605-019-04395-7

[51] Bertrand LR , Abdallah M , Espinel Y , Calvet L , Pereira B , Ozgur E , et al. A case series study of augmented reality in laparoscopic liver resection with a deformable preoperative model. Surg Endosc. 2020;34(12):5642–8. 32691206 10.1007/s00464-020-07815-x

[52] Pelanis E , Teatini A , Eigl B , Regensburger A , Alzaga A , Kumar RP , et al. Evaluation of a novel navigation platform for laparoscopic liver surgery with organ deformation compensation using injected fiducials. Med Image Anal. 2021;69:101946. 33454603 10.1016/j.media.2020.101946

[53] Zhang W , Zhu W , Yang J , Xiang N , Zeng N , Hu H , et al. Augmented reality navigation for stereoscopic laparoscopic anatomical hepatectomy of primary liver cancer: preliminary experience. J Front Oncol. 2021;25(11):663236. 10.3389/fonc.2021.663236 PMC8027474 33842378

[54] Saito Y , Sugimoto M , Imura S , Morine Y , Ikemoto T , Iwahashi S , et al. Intraoperative 3D hologram support with mixed reality techniques in liver surgery. Ann Surg. 2020;271(1):e4–7. 31425293 10.1097/SLA.0000000000003552

[55] Saito Y , Sugimoto M , Morine Y , Imura S , Ikemoto T , Yamada S , et al. Intraoperative support with three‐dimensional holographic cholangiography in hepatobiliary surgery. Langenbecks Arch Surg. 2021. Online ahead of print. 10.1007/s00423-021-02336-0 34557939

[56] Aoki T , Koizumi T , Sugimoto M , Murakami M . Holography‐guided percutaneous puncture technique for selective near‐infrared fluorescence‐guided laparoscopic liver resection using mixed‐reality wearable spatial computer. Surg Oncol. 2020;35:476–7. 33113479 10.1016/j.suronc.2020.10.013

[57] Kitaguchi D , Takeshita N , Matsuzaki H , Oda T , Watanabe M , Mori K , et al. Automated laparoscopic colorectal surgery workflow recognition using artificial intelligence: Experimental research. Int J Surg. 2020;79:88–94. 32413503 10.1016/j.ijsu.2020.05.015

[58] Yamazaki Y , Kanaji S , Matsuda T , Oshikiri T , Nakamura T , Suzuki S , et al. Automated surgical instrument detection from laparoscopic gastrectomy video images using an open source convolutional neural network platform. J Am Coll Surg. 2020;230(5):725–32.e1. 32156655 10.1016/j.jamcollsurg.2020.01.037

[59] Tokuyasu T , Iwashita Y , Matsunobu Y , Kamiyama T , Ishikake M , Sakaguchi S , et al. Development of an artificial intelligence system using deep learning to indicate anatomical landmarks during laparoscopic cholecystectomy. Surg Endosc. 2021;35(4):1651–8. 32306111 10.1007/s00464-020-07548-x PMC7940266

[60] Kitaguchi D , Takeshita N , Matsuzaki H , Hasegawa H , Honda R , Teramura K , et al. Computer‐assisted real‐time automatic prostate segmentation during TaTME: a single‐center feasibility study. Surg Endosc. 2021;35(6):2493–9. 32430531 10.1007/s00464-020-07659-5

[61] Kitaguchi D , Takeshita N , Matsuzaki H , Igaki T , Hasegawa H , Ito M . Development and validation of a 3‐dimensional convolutional neural network for automatic surgical skill assessment based on spatiotemporal video analysis. JAMA Netw Open. 2021;4(8):e2120786. 34387676 10.1001/jamanetworkopen.2021.20786 PMC8363914

[62] Nazir A , Cheema MN , Sheng B , Li P , Li H , Yang P , et al. SPST‐CNN: Spatial pyramid based searching and tagging of liver's intraoperative live views via CNN for minimal invasive surgery. J Biomed Inform. 2020;106:103430. 32371232 10.1016/j.jbi.2020.103430

